# HIV Screening During Pregnancy in a U.S. HIV Epicenter

**DOI:** 10.1155/2020/8196342

**Published:** 2020-05-07

**Authors:** Alec Szlachta-McGinn, Alexandra Aserlind, Lunthita Duthely, Sean Oldak, Ruchi Babriwala, Emily Montgomerie, JoNell Potter

**Affiliations:** Department of Obstetrics and Gynecology, University of Miami Miller School of Medicine, Miami, FL, USA

## Abstract

**Background:**

The CDC and ACOG have issued guidelines for HIV screening in pregnancy for patients living in areas with high prevalence of HIV in order to minimize perinatal vertical transmission. There is a lack of data examining providers' compliance with these guidelines in at-risk patient populations in the United States.

**Objective:**

To evaluate if HIV screening in pregnant women was performed according to guidelines at a large, urban, tertiary care medical center in South Florida. *Study Design*. A retrospective review was performed on 1270 prenatal and intrapartum records from women who delivered a live infant in 2015 at a single institution. Demographic and outcome data were chart abstracted and analyzed using arithmetic means and standard deviations.

**Results:**

Of the 1270 patients who met inclusion criteria, 1090 patients initiated prenatal care in the first or second trimester and delivered in the third trimester. 1000 (91.7%) patients were screened in the first or second trimester; however, only 822 (82.2%) of these were retested in the third trimester during prenatal care. Among the 178 patients lacking a third trimester test, 159 (89.3%) received rapid HIV testing upon admission for delivery. Of the 1090 patients who initiated prenatal care in the first or second trimester and delivered in the third trimester, 982 (90.1%) were screened in accordance with recommended guidelines. Of the 1270 patients initiating care in any trimester, 24 (1.9%) had no documented prenatal HIV test during prenatal care, however 22 (91.7%) had a rapid HIV test on admission for delivery. Two (0.16%) patients were not tested prenatally or prior to delivery.

**Conclusion:**

Despite 99.8% of women having at least one HIV screening test during pregnancy, there is room for improvement in routine prenatal screening in both early pregnancy and third trimester prior to onset of labor in this high-risk population.

## 1. Introduction

In 2017, Florida ranked first in any state in the United States (U.S.) in new human immunodeficiency virus (HIV) infections with 22.9 cases per 100,000 person years [1]. South Florida contains the highest number of HIV diagnoses in the state per year, with the total number of new diagnoses in these counties greater than in most states in the U.S. [2]. Additionally, a study of perinatal HIV infection in the U.S. demonstrated that Florida was one of five southern states that accounted for a large portion of perinatal HIV infection in 2013 [3].

Perinatal HIV transmission can occur during pregnancy, labor and delivery, or postdelivery during breastfeeding. Since the mid-1990s, mother-to-child HIV transmission rates have decreased dramatically, owing to the Centers for Disease Control and Prevention (CDC) initiative to offer HIV screening to all pregnant women, to treat HIV-infected women with antiretroviral (ARV) medication during pregnancy, and to discourage breastfeeding by HIV-positive mothers [3], [4]. With the goal of reducing mother-to-child HIV transmission to 1% or less, the CDC made the following revisions to the recommendations regarding HIV screening in pregnancy in 2006 for high-risk populations (HIV incidence greater than 17 cases per 100,000 person years): universal opt-out HIV screening to all pregnant women in early pregnancy, a repeat test in the third trimester for at-risk women, rapid HIV testing at labor and delivery for women without a second test in the third trimester or unknown HIV status, and immediate initiation of ARV therapy during labor if the rapid test result is positive while awaiting confirmatory test results [5]. These recommendations are supported by the American College of Obstetricians and Gynecologists (ACOG) [6]. In addition to geographic prevalence of HIV, other risk factors that place women at high risk of acquiring HIV in pregnancy include women who have been diagnosed with another sexually transmitted infection in the past year; injection drug users; those who have sex with injection drug users; those who exchange sex for money or drugs; and women who have a new sexual partner, multiple sexual partners, or a sexual partner infected with HIV during pregnancy [6]. Finally, many states, including Florida, have updated their HIV screening during pregnancy laws to reflect the recommendations of the CDC and ACOG [7], [8]. In summary, patients are screened as early as possible in pregnancy, and repeat screening is offered during the third trimester. In the absence of a third trimester HIV screening test, testing on admission for delivery is performed. Patients may opt-out of HIV screening during pregnancy in Florida.

The objective of this study was to evaluate if providers perform HIV screening during pregnancy according to guidelines recommended by both the CDC and ACOG, as well as Florida law, at Jackson Memorial Hospital (JMH), a large, urban, tertiary care academic medical center in South Florida. All pregnant women seeking prenatal care at this nonprofit, publically funded institution are deemed to be at risk for HIV infection by living in an area with a high prevalence of HIV. This underscores the importance of ensuring HIV screening is performed according to guidelines in this high-risk patient population, and previous studies have shown that HIV screening in pregnancy is not universal [3], [9]. To the authors' knowledge, there is a paucity of data that examines HIV screening in pregnancy for at-risk women who live in an area with high HIV prevalence in the United States.

## 2. Materials and Methods

Jackson Memorial Hospital has a large, underinsured, and underserved patient population where many high-risk patients receive prenatal care in the obstetrics clinics and deliver at the hospital, which houses a neonatal intensive care unit that accepts newborns beginning at 23 weeks of gestation. Approximately 5-6% of women receiving prenatal care at JMH annually are HIV carriers. Known HIV-positive women receive prenatal care at the primary HIV clinic at JMH. There are no HIV-positive women who receive prenatal care in the regular obstetrics clinic. HIV screening during pregnancy at JMH follows the aforementioned guidelines. A third trimester HIV test is defined as one that occurs at any time during the third trimester of prenatal care. Ordering HIV screening tests in pregnancy at JMH requires the provider to manually enter the order in the hospital's electronic medical record (EMR) at the appropriate clinic visit, as HIV testing is not part of an order set within the EMR.

The year 2015 was chosen for this study because this was the first full year after ACOG updated trimester definitions according to gestational age [10]. We felt that due to these changes, there may be a subset of providers who were using old definitions of trimesters in pregnancy and therefore not properly screening pregnant women for HIV. Live births at Jackson Memorial Hospital between January 1, 2015, and December 31, 2015, were identified using current procedure terminology (CPT) codes from 2015. Women who initiated prenatal care with an unknown or documented negative HIV status and delivered a liveborn infant at JMH during the study period were included. Women less than 18 years of age at the time of delivery, those who opted out of HIV testing, and those who received prenatal care at outlying facilities were excluded from the study. Prenatal care was defined as at least one medical care visit during the pregnancy. A retrospective review was performed on 1270 records meeting these criteria. Data collection started in November 2015 and ended in April 2016. Institutional Review Board approval at the University of Miami was obtained prior to data extraction (IRB #20150697).

A team of four researchers reviewed the records in the hospital's electronic medical record. After confirmation of a live birth, the team searched the EMR for prenatal clinic notes in the obstetrics clinic. The first prenatal record for every patient was reviewed to exclude known HIV-positive women. To evaluate whether or not screening was carried out according to the guidelines (HIV screening upon initiation of prenatal care with repeat screening offered during the 3^rd^ trimester or admission to labor and delivery), the following data were collected on patients meeting the inclusion criteria from the prenatal clinic notes and laboratory review: gestational age at first and last prenatal visit, gestational age at first and second (or last) HIV test, result of first and second (or last) HIV test, and whether or not a rapid HIV test was performed on admission to labor and delivery. Demographical data, including patients' age, race, education level, and gestational age at delivery, were obtained from available records.

The data were deidentified on the data collection sheet, and a study number was assigned to each record. A master list with patient identifiers and the corresponding study numbers was only available to the research team and kept separately from the data in order to facilitate repeat chart reviews at a later date. After initial data collection, a repeat chart review was performed by two of the four researchers on each data point to confirm accuracy and consistency of the data entered. The data were analyzed using proportions, arithmetic means, and standard deviations. Data analysis was repeated two more times by separate researchers to confirm accuracy and consistency.

The Bio-Rad's GS HIV Combo Antigen/Antibody enzyme immunoassay was used at this institution in 2015 for prenatal screening of HIV. The OraQuick Advance Rapid HIV-1/2 Antibody Test was used at this institution in 2015 for rapid HIV screening on labor and delivery. Trimesters were defined according to the ACOG definition in 2014 as follows: first trimester from 0 weeks to 13 weeks 6 days, second trimester from 14 weeks 0 days to 27 weeks 6 days, and third trimester from 28 weeks 0 days to delivery [10].

HIV screening tests during prenatal care is often bundled with other tests to optimize patient adherence with scheduled appointments and to reduce the frequency of prenatal care and laboratory visits. Therefore, in this study, we determined that a second HIV screening test performed as early as 25-week EGA was compliant with repeat third trimester HIV screening guidelines recommended by both the CDC and ACOG.

## 3. Results

**Figure 1:**
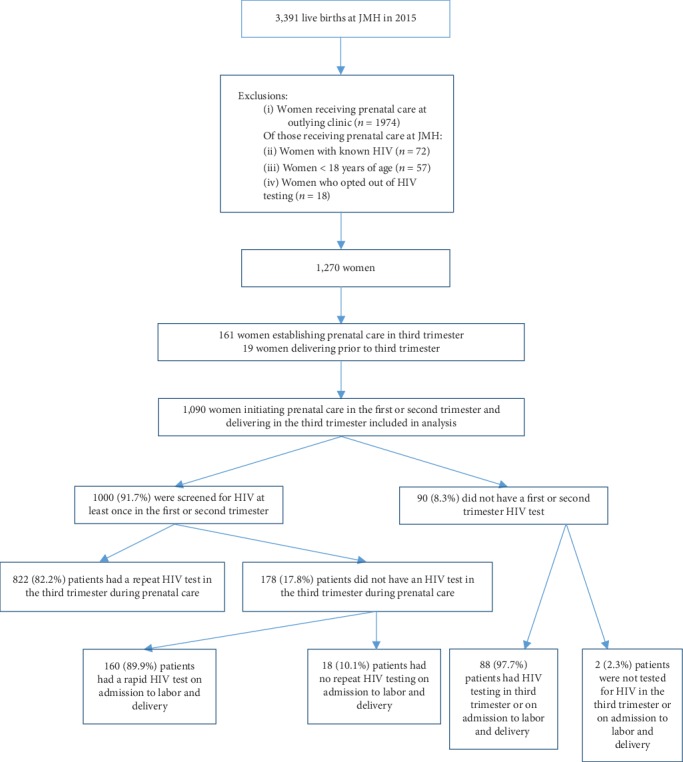
Flow diagram illustrating inclusion and exclusion criteria of patients in this study. Of those included in the final analysis, prenatal HIV screening rates are shown according to CDC and ACOG guidelines.

Of the 3391 deliveries in 2015 at the hospital under study, 1270 patients met inclusion criteria for this study (Figure 1). Characteristics of the 1270 study participants are shown in Table 1. The mean age of the patients at delivery was 29.5 years (SD ± 6.6). The majority of the patients identified as Hispanic (*n* = 807, 63.5%), 320 (25.2%) identified as black non-Hispanic, 71 (5.6%) identified as white non-Hispanic, and 72 (5.7%) identified as other or unknown. Of the 588 patients for which education level was identifiable during chart review, the majority of patients attained high school education or above (*n* = 366, 70.9%), with 84 (14.3%) not continuing education beyond 8^th^ grade. Most patients (*n* = 1163, 91.6%) delivered at an estimated gestational age (EGA) of 36 weeks or greater, 100 (7.8%) of patients delivered between 24 and 35 weeks, and 7 (0.6%) of patients delivered at an EGA of less than 24 weeks.

Of the 1270 who met the inclusion criteria, 617 (48.6%) initiated prenatal care in the first trimester. Of these, 520 (84.3%) received the first HIV screening test in the first trimester, while 76 (12.3%) were not screened until the second trimester. 17 (2.8%) were not screened for HIV until the third trimester. 4 (0.6%) patients who initiated prenatal care in the first trimester did not receive an HIV screening test at any time during prenatal care (Table 2).

Of the 1270 qualifying patients, 492 (38.7%) initiated prenatal care in the second trimester. Of these, 17 (3.4%) had been previously screened for HIV at either an outside clinic or at a laboratory-only visit at JMH with documentation of a previous HIV negative result, 404 (82.1%) had a first HIV screening test performed at initiation of prenatal care in the second trimester, and 57 (11.6%) were not screened for HIV until the third trimester. 14 (2.8%) patients who initiated prenatal care in the second trimester did not receive an HIV screening test at any time during prenatal care (Table 2).

Of the 1270 qualifying patients, 161 (12.7%) initiated prenatal care in the third trimester. Of these, 10 (6.2%) had been previously screened for HIV at either an outside clinic or at a laboratory-only visit at JMH with documentation of an HIV-negative result. The remaining 145 (90.1%) had the first HIV screening test performed at initiation of prenatal care during the third trimester. Six (3.7%) patients who initiated prenatal care in the third trimester did not receive an HIV screening test during prenatal care (Table 2).

In total, of the 1270 qualifying patients, 24 (1.9%) of patients did not have documentation of having received a single HIV screening test during prenatal care. Of those who did not have HIV testing prenatally, 22 (91.7%) had a rapid HIV test on labor and delivery only. Out of the 1270 total patients, 2 (0.16%) did not have HIV screening during prenatal care or at time of delivery (Table 2).

Next, we used trimester at initiation of prenatal care to stratify rates of primary and repeat HIV screening. 1000 of the 1090 (91.7%) patients who initiated care in the first or second trimester and delivered at 28 weeks or later were screened for HIV in the first or second trimester. Of these, 822 (82.2%) had a documented second HIV screening test during prenatal care. The remaining 178 (17.8%) did not have a second documented HIV screening test during prenatal care; however, 160 (89.9%) had documentation of a rapid HIV test order on admission to labor and delivery. A total of 159 had a completed test. In 1 case, the rapid HIV test was ordered; however, the test was not conducted. Of the 18 (10.1%) patients with initial first or second trimester HIV screen but no third trimester or labor admission screen, no additional information was found (Table 3). Of the 90 patients (8.3%) who initiated care in the first or second trimester and delivered at 28 weeks or later who were not screened for HIV in the first or second trimester, 88 (97.7%) were tested in either the third trimester or on admission to L&D, and 2 (2. 3%) did not receive HIV screening at all (Table 2).

There was one positive HIV screening test result in this study. This positive result was identified on a rapid HIV screening test on admission to labor and delivery; however, confirmatory testing was negative (Table 3). There were no cases of HIV seroconversion in this study (data not shown).

## 4. Discussion

Perinatal HIV transmission is preventable with appropriate maternal HIV screening and early initiation of ART if the screening test is positive. Women should receive one HIV screening test as early as possible during pregnancy to establish baseline HIV status, and a second HIV screening test during the third trimester, as some women remain sexually active during pregnancy, placing them at a greater risk for seroconversion [5, [6].

Despite the vast majority of states, including Florida, enacting statutes consistent with CDC recommendations [7], [8], multiple studies have shown that HIV screening of pregnant women is not universal. Unlike the near-universal screening rate for syphilis and hepatitis B virus, a recent study by Ross et al. in 2015 estimated that 1 in 6 pregnant women with prenatal care do not receive at least 1 HIV screening test [9]. Another study by Taylor et al. of perinatal HIV infection among infants born in the United States demonstrated that in 2013, the incidence of perinatal HIV transmission was 1.75 per 100,000 live births, 75% higher than the CDC's goal of 1 per 100,000 live births [3].

In our study at a large, urban, tertiary care medical center in South Florida, HIV screening during pregnancy was performed according to guidelines in 90% of patients who initiated prenatal care in the first or second trimester. We also found that 1.9% were never screened for HIV during their prenatal care, and 0.16% were neither screened for HIV during prenatal care nor upon admission for delivery. The remaining 8% of patients were screened for HIV in pregnancy at intervals inconsistent with current guidelines. Moreover, we found that a similar portion of women is tested at the initiation of prenatal care: 84.3% in first trimester and 82.1% in second trimester.

A recent study by Scott et al. in 2017 identified late initiation of ART as a missed opportunity in the obstetrical care in women living with HIV that led to perinatal HIV infection between 2002 and 2009. Scott et al. found that other missed opportunities including poor adherence to ART, poor control of viral load, and prolonged duration of rupture of membranes over 4 hours increased the risk of mother-to-child HIV transmission. The study also found that women frequently had more than one missed opportunity [11]. Other published literatures identified the same missed opportunities in preventing perinatal HIV transmission, in addition to the lack of elective cesarean delivery [12]–[15].

It should be noted that while we deliberately chose to exclude minors from this study, this patient population should be screened for HIV in pregnancy according to the same guidelines as adult women. Additionally, we chose to exclude women who received prenatal care at private clinics outside of the Jackson Health System for the following reasons. First, we desired to investigate how compliant our institution is with HIV screening in pregnancy, as any deficiencies could be addressed with an internal quality improvement study to increase compliance. Second, we felt that restricting the study to women who received prenatal care and delivered at JMH would provide a stronger data analysis given that we have their complete prenatal records for review. Prenatal care documentation from outside private clinics was not reliably available for a retrospective review, which would have led to missing data and therefore introduced unwanted bias into our results.

A limitation of this study is the limited generalizability to low-risk patient populations. Our study is very specific to a patient population deemed to be at an increased risk for transmission of HIV during pregnancy, which we feel is important for other high-risk populations in the United States and the world. However, we acknowledge that repeat HIV screening in the third trimester may not be warranted for all pregnant women. Another limitation is the possibility of poor documentation by obstetricians and nursing staff in the obstetrics clinics and on the labor and delivery. Poor documentation of HIV orders, test results, and reasons for declining HIV screening tests leads to overestimation of the number of cases in which HIV screening during pregnancy was not followed according to guidelines and state law. In fact, multiple studies have shown poor documentation of HIV status in prenatal records both before and after the CDC issued its recommendations regarding HIV screening in pregnancy in 2006 [16]–[20]. Other limitations of this study that overestimate the number of cases in which HIV screening in pregnancy was not followed according to guidelines include inadequate prenatal care, whereby there are too few visits for the provider to order an HIV screening test; poor patient compliance with scheduled prenatal and laboratory visits; delivery early in the third trimester before repeat HIV screening could be completed; and providers following previous trimester definitions when ordering HIV screening tests (for example, the second trimester spanning from 13 weeks 0 days to 23 weeks 6 days, and the third trimester beginning at 24 weeks). Finally, our data examines HIV screening of our population as a whole. It does not compare HIV screening rates by demographic data, such as race and education level.

While a small fraction of women did not receive HIV screening according to the CDC and ACOG guidelines, this study has shown that most of our patients at JMH receive at least one HIV screening test in pregnancy, and nearly all are tested a second time in the third trimester or at the time of delivery. Provider noncompliance with prenatal HIV screening represents potential missed opportunities for diagnosis of HIV and early initiation of ART in order to eliminate perinatal HIV transmission. Therefore, provider education and awareness of HIV screening recommendations set forth by the CDC and ACOG are crucial to ensure appropriate patient selection and optimal screening for at-risk pregnant women. Providers should familiarize themselves with the HIV incidence and prevalence in their respective geographical areas, which can be obtained through public health records. Additionally, obtaining a nonbiased social and sexual health history during pregnancy can identify high-risk women in whom a repeat HIV screening test in the third trimester is recommended.

This study only evaluated whether providers perform HIV screening in pregnancy according to guidelines at our institution. Our data show that nearly 10% of patients did not receive HIV screening according to guidelines for high-risk pregnant women during prenatal care prior to delivery. This finding may be secondary to noncompliance with third trimester prenatal visits arising from the barriers and challenges associated with access to care and low health literacy in our patient population. Assessing these risk factors, as well as the impact that race and education level has on HIV screening rates, is one important future direction that we plan to study. Finally, we plan perform a follow-up study to see if HIV screening in this high-risk population has changed over time.

## Figures and Tables

**Table 1:** Characteristics of the patient population. Age at delivery, race/ethnicity, and estimated gestational age were identified for all 1270 patients. Education level was identified for 588 patients of the cohort.

Demographic data	
Age	Mean ± SD
Age at delivery (in years)	29.5 (±6.6)
Race/ethnicity	*N* (%)
Black, non-Hispanic	320 (25.2)
Hispanic (all races)	807 (63.5)
White, non-Hispanic	71 (5.6)
Other/unknown	72 (5.7)
Education	(*n* = 588) *N* (%)
8^th^ or less	84 (14.3)
Did not graduate high school	87 (14.8)
High school diploma/GED	236 (40.1)
Some college	86 (14.6)
College degree	39 (6.6)
Graduate level education	5 (0.9)
Unspecified	51 (8.7)
EGA at delivery (weeks)	*N* (%)
<24	7 (0.6)
24-27	12 (0.9)
28-35	88 (6.9)
≥36	1163 (91.6)

**Table 2:** Trimester of initiation of prenatal care vs first HIV screening test. The trimester during which the first HIV test was documented was identified and compared to the trimester during which prenatal care was initiated for all patients meeting inclusion criteria. HIV screening occurring in the trimester of initiation of prenatal care signifies early HIV screening in pregnancy. PNC = prenatal care: 1270.

Timing of the first HIV test in pregnancy				
Timing of HIV test		Trimester in which prenatal care began		
		1^st^ trimester	2^nd^ trimester	3^rd^ trimester
(*n* = 1270)		(*N* = 617)	(*N* = 492)	(*N* = 161)
Previously screened			17 (3.4%)	10 (6.2%)

Trimester of first HIV test	First	520 (84.3%)		
	Second	76 (12.3%)	404 (82.1%)	
	Third	17 (2.8%)	57 (11.6%)	145 (90.1%)

No HIV test during PNC		4 (0.6%)	14 (2.9%)	6 (3.7%)

Rapid test in L&D only		2	14	6

No HIV test during PNC and no rapid test on labor and delivery		2	0	0

**Table 3:** Third trimester/second HIV screening test and rapid HIV test at delivery in eligible patients. Repeat HIV screening was identified in patients who initiated prenatal care in 1^st^ or 2^nd^ trimester, and delivered during the third trimester (≥28 weeks) (*n* = 1090). In those without repeat HIV screening during prenatal care, we identified whether rapid HIV testing at delivery was performed.

Timing of the second HIV test in pregnancy	
1^st^/2^nd^ trimester PNC, delivered 3^rd^ trimester	*n* = 1090
1^st^ prenatal HIV testing: 1^st^/2^nd^ trimester	1000/1090 (91.7%)
Third trimester/second HIV test:	822/1000 (82.2%)
No third trimester/no second HIV test in PNC:	178/1000 (17.8%)
Patients delivering in third trimester with no third trimester HIV test or second HIV test:	*n* = 178
Rapid HIV test documented at delivery	160 (89.9%)
Rapid HIV completed:	159 (89.3%)^∗^
Rapid HIV test ordered, not done:	1 (0. 6%)
Rapid HIV test not performed at delivery:	18 (10.1%)

^∗^Includes one rapid test HIV positive on labor and delivery; confirmatory testing negative.

## Data Availability

The retrospective data used to support the findings of this study are included within the article.
